# Intramedullary nailing of proximal humerus fractures does not achieve superior functional results to non-operative treatment in the long term

**DOI:** 10.1007/s00402-024-05421-3

**Published:** 2024-08-06

**Authors:** Leopold Henssler, Christian Pfeifer, Moritz Riedl, Teresa Schneider, Miriam Kobeck, Volker Alt, Arne Berner, Maximilian Kerschbaum, Lisa Klute

**Affiliations:** 1 Department of Trauma Surgery, University Hospital Regensburg, Franz-Josef-Strauss-Allee 11, 93053 Regensburg, Germany; 2 Department of Trauma and Hand Surgery, Altoetting, Vinzenz-von-Paul-Straße 10, 84503 Altoetting, Germany; 3 Department of Trauma Surgery, Bad Neustadt, Von-Guttenberg-Straße 11, 97616 Bad Neustadt an der Saale, Germany

**Keywords:** Humeral head, Shoulder, Intramedullary nailing, Conservative, Fracture, Geriatric

## Abstract

**Introduction:**

Non-operative treatment (NOT) of proximal humerus fractures (PHF) has regained significance due to recent evidence. Additionally, positive outcomes of plate osteosynthesis and fracture arthroplasty prompt a reassessment of the role of intramedullary nailing (IMN). While favorable short and medium-term results have been documented following IMN, little is known regarding functional outcomes and quality of life in the long-term.

**Methods:**

Data from 180 patients with dislocated PHF of Neer types III, IV and V, treated at our level-I trauma center between 2004 and 2014 using IMN or NOT therapy, were scanned. Patients were re-evaluated after a minimum of 5 years to assess functional outcomes (age- and sex-adapted Constant Score, QuickDASH), quality of life (SF12), and complications or reoperations.

**Results:**

Out of the initially identified 180 patients, 51 were unavailable for follow-up (FU) and 71 had deceased during the FU period. Functional outcomes and quality of life was, therefore, assessed in 58 patients (30 IMN, 28 NOT) with an average age at injury of 68 years after a mean FU time of 10.3 ± 3.4 years. Epidemiological patient characteristics did not exhibit significant differences between the two groups (*p* > .05). The functional outcome assessed by age- and sex-adapted Constant Score (NOT: 74 ± 28; IMN: 68 ± 24; *p* = .438), QuickDASH (NOT: 25 ± 27; IMN: 31 ± 23; *p* = .374) or quality of life using the SF12 (*p* > .05) revealed no significant disparities in long-term outcomes between the treatment groups. 10 of 30 patients in the IMN group underwent surgical revision to address complications, exceeding mere implant removal. Conversely, no patient in the NOT group underwent a revision surgery during the FU period.

**Conclusions:**

In the long-term, functional and quality of life-related outcomes of IMN did not diverge significantly from those of NOT, while causing a higher incidence of follow-up interventions.

## Highlights

**•** Comparing intramedullary nailing to non-operative treatment of Neer type III - V proximal humerus fractures, there was no significant difference in long-term functional outcome.
**•** Revision rates were significantly higher in the surgically treated patient group.
**•** Irrespective of the treatment modality long-term functional outcomes were significantly worse in fractures with more than two key fragments according to the Codman Classification.

## Introduction

Proximal humerus fractures (PHF) rank as the third most common fracture site among adults, following fractures of the proximal femur and distal radius, with increasing incidence [1]. These fractures frequently lead to functional impairment, a concern accentuated by their prevalence in the elderly population [1]. As demographic shifts contribute to an aging society, the relevance of proximal humerus fractures is anticipated to further rise. Despite the increasing incidence, determining the optimal approach for treating these fractures remains a dynamic area of investigation.

While surgical fixation of PHF has demonstrated improved outcomes in the short-term, recent large controlled trials have revealed comparable functional results with non-operative treatment [2], especially in the mid- to long-term. Consequently, non-operative treatment (NOT) has gained popularity over the past decade [3]. Concurrently, the use of intramedullary nailing (IMN) for operative treatment has diminished by 80%, owing to favorable outcomes with open reduction and plate fixation, as well as the ascendancy of reverse arthroplasty [3]. Despite accumulating evidence, uncertainty persists among clinicians in selecting the most suitable treatment for individual patients [4]. Surgical treatment by head preserving procedures yields beneficial results in early recovery [5] but is accompanied by an elevated complication rate compared to non-operative treatment [6], especially when ORIF by plate is performed [2, 7]. Thus, intramedullary nailing remains a common choice in vulnerable patient groups like geriatric or severely injured patients, where a rapid recovery with minimal invasiveness is desired.

However, the existing literature comparing IMN with NOT predominantly focuses on short to medium-term results. Consequently, it remains unclear whether minimally invasive IMN can extend its early recovery benefits to long-term functional outcomes and potentially prevent patients from undergoing revision surgeries or converting to total shoulder arthroplasty. Therefore, the objective of this study is to investigate long-term patient-reported outcomes after intramedullary nailing and non-operative treatment while comparing complications and revision rates.

## Methods

### Patient collective

For the analysis, we identified all patients treated for proximal humerus fractures at our level-I trauma center between 2004 and 2014. A total of 522 fractures, including 2-part, 3-part, and 4-part fractures, were initially screened for primary analysis. The fractures were categorized according to Neer’s classification [8] and only displaced proximal humerus fractures with 1 cm displacement or 45° angulation of key fragments were included for further analysis (Neer II-VI), whereas undisplaced fractures (Neer type I) were primarily excluded. Patients undergoing treatment other than head-preserving approaches (e.g., primary treatment by shoulder arthroplasty), those under 18 years old, and those with pathologic fractures were excluded. Additionally, fractures of the anatomical neck (Neer type II), fracture dislocations (Neer type VI) and isolated fractures of the tuberosities (Neer IV or V/2-part-fractures) were excluded due to their increased risk of avascular necrosis of the humeral head and limited suitability for intramedullary nailing [9–11], respectively. Consequently, only Neer type III, type IV, and type V fractures were considered. In the subsequent step, patients undergoing non-operative treatment or surgical treatment by intramedullary nailing were identified.

**Fig. 1:**
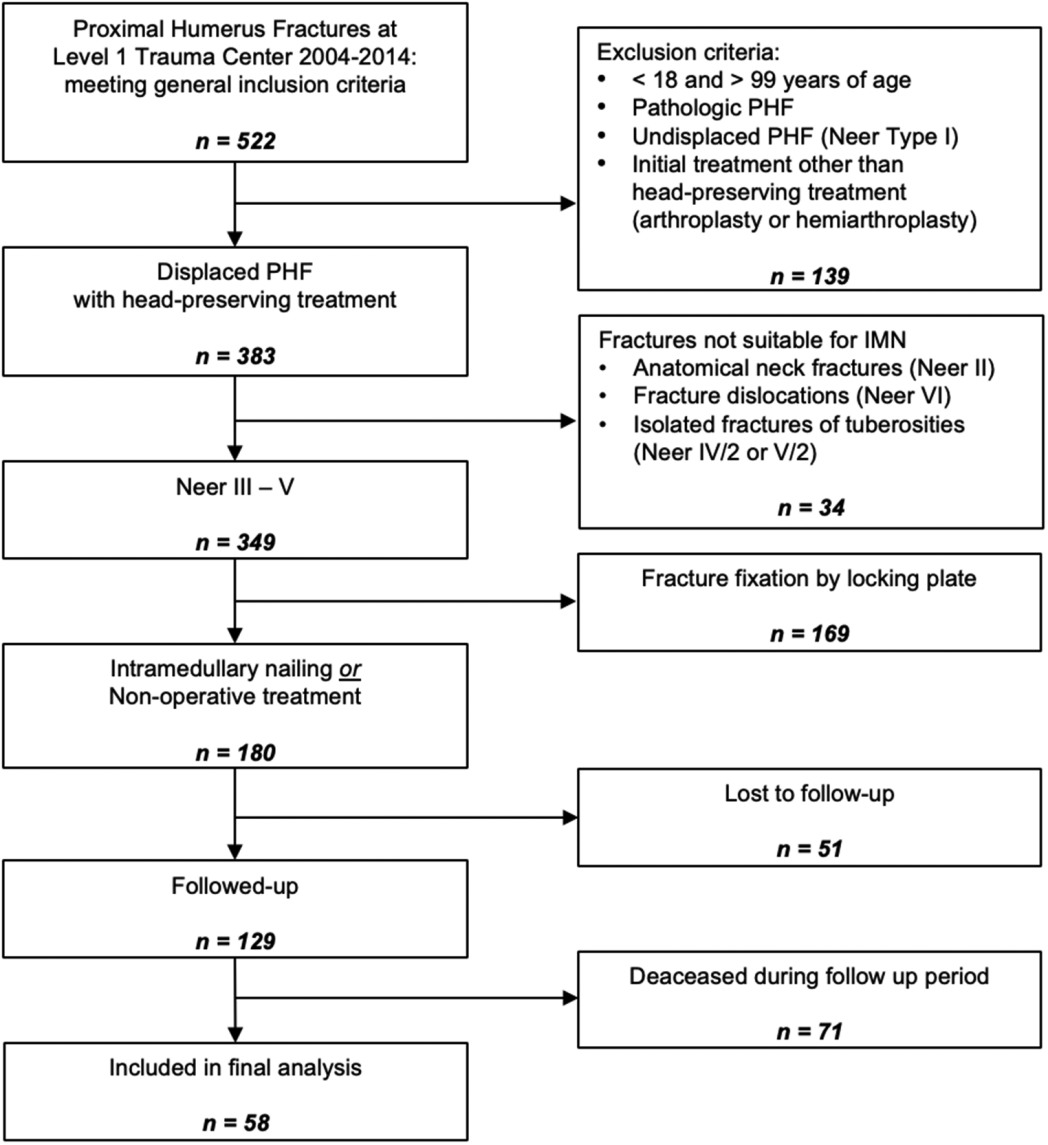
Flow chart of patient inclusion and exclusion. The final analysis includes only patients with Neer type III – V fractures treated by intramedullary nailing or non-operative treatment and with available follow up data after a minimum period of 5 years

After a minimum follow-up period of 5 years, 180 patients were contacted for the assessment of functional outcomes and quality of life via telephone interviews. Before functional assessment, all participants gave their informed consent. Complete functional and clinical follow-up data were obtained from 58 patients, while 51 patients were lost to follow-up, and 71 had died within the follow-up period (see Fig. 1). The study protocol and procedures were approved by the local Ethics Committee of the University (Nr. 20-1733-101) prior to study initiation.

### Surgical treatment by intramedullary nailing (IMN)

**Fig. 2:**
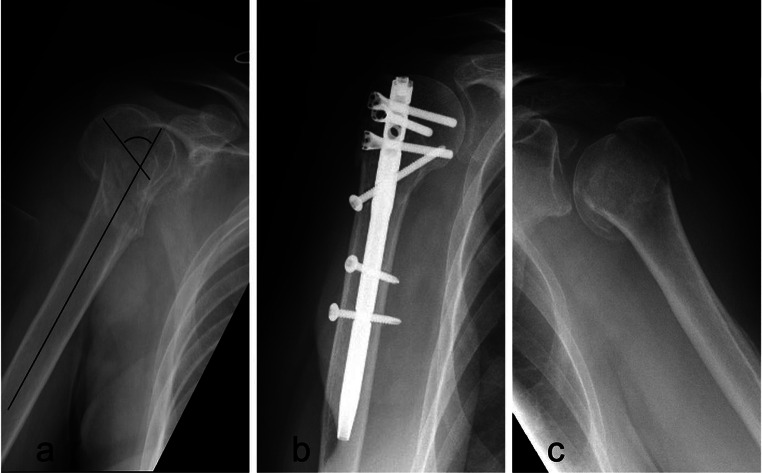
provides radiographs of each treatment modality. **(a)** A 69-year-old female patient presented with a 3-part fracture of her dominant right shoulder with significant valgus displacement of the head, displacement of the greater tuberosity (Neer type IV) and comminution of the calcar. **(b)** She underwent treatment with intramedullary nailing (IMN) using MultiLoc (DePuy Synthes). **(c)** A 77-year-old female patient from a nursing home presented with a varus-displaced impacted 3-part fracture (Neer type IV) and received non-operative treatment

Treatment decisions were primarily influenced by factors such as age, comorbidities, associated orthopaedic injuries requiring surgery, and the specific fracture characteristics such as translation type displacement and comminution of the calcar region. Notably, patients with a greater need for self-independence typically underwent surgical treatment. Operative treatment was conducted with patients in the beach chair position. Before sterile washing and covering of the surgical site, the feasibility of a closed reduction of the fracture was verified by X-ray fluoroscopy. Subsequently, the intramedullary proximal humerus nail (Targon PH, Aesculap AG, Tuttlingen, Germany; MultiLoc, DePuy Synthes, Massachusetts, USA) was implanted according to the manufacturer’s recommended procedures (see Fig. 2). Postoperatively, the affected shoulder was immobilized with a Gilchrist bandage for 7 to 14 days, gradually reducing the wearing time over the first two weeks. Functional therapy, starting with pendulum exercises at the first to the second postoperative day, was gradually increased, and active-assistive shoulder motion exercises were added after several days.

### Non-operative treatment (NOT)

Patients receiving non-operative treatment had their affected shoulder immobilized with a Gilchrist bandage for 7 to 14 days. After 5 days, functional therapy, starting with pendulum exercises, commenced. Beginning after 14 days, passive and active-assistive shoulder motion exercises were added under physiotherapeutic guidance to self-exercises with increasing range of motion. Later in the rehabilitation program, pain-adapted self-assisted active anteversion and abduction exercises were implemented. During the first six weeks of rehabilitation, X-ray controls were scheduled after 3–5 days, 10–14 days, and 17–21 days. Loading of the arm was prohibited for 6 weeks and then gradually increased after an additional X-ray control. Pain-adapted analgesic therapy was provided throughout the entire period.

### Epidemiologic and functional assessment

In addition to recording epidemiologic patient characteristics (sex, age, follow-up time), comorbidities (Charlson Comorbidity Index, CCI), and additional injuries, all fractures were subclassified according to Codman’s classification of main fragments and Neer’s classification.

In all available patients, shoulder function was assessed via telephone interviews by trained medical staff using an adapted version of the Constant-Murley score (CS) for patient self-assessment, which was developed and validated by Böhm et al. [12]. The score combines patient-reported subjective pain (15 points) and activities of daily living (20 points) with objective parameters range of motion (40 points) and muscle strength (25 points) that can be measured by patients using standard household items. To ensure that the scores were collected correctly, patients were supported by healthcare professionals over the phone. As part of the questionnaire, the ROM was queried in categorical variables by asking about the ability to reach certain positions. To mitigate potential confounders, the sex- and age-adapted Constant Score (aCS) was calculated. Limitations of the affected arm in daily activities were evaluated using the QuickDASH questionnaire, which includes 11 questions on abilities in daily activities and sports. Furthermore, quality of life was estimated using the Short-Form 12 questionnaire (SF-12) and its subscales for physical health (SF-12-PCS) and mental health (SF-12-MCS).

### Statistical analysis and reporting of results

Statistical analysis was carried out using SPSS software package version 25 (SPSS Inc., Chicago, IL, USA). Distribution of variables was testes by Kolmogorov–Smirnov normality test. Descriptive data are expressed in terms of mean ± standard deviation (range). For comparison of groups, the independent t-test was used for continuous normally distributed variables, the Mann-Whitney-U-Test for independent non-metric variables and chi-squared test was used for comparison of the distribution of patient characteristics. The level of significance was set at *p* = .05 for all statistical tests. All results were reposted according to the STROBE guidelines.

## Results

### Patient characteristics

Out of the initial 180 patients eligible for the study, 51 were unavailable for follow-up, and an additional 71 of the remaining 129 patients had died during the follow-up period (see Fig. 1). The cohort of patients who had died within the follow-up period was significantly older (at the time of injury) and had a significantly higher proportion of seriously comorbid patients than those patients of which outcome assessment could be recorded. Among the patients lost to follow-up, the proportion of men was significantly higher, but they did not differ significantly from the patients available for follow-up in terms of age, distribution of treatments and proportion of comorbid patients (see Table 1).

**Table 1:** Patients who died during the follow-up period and were therefore not available for evaluation were significantly older and included significantly more patients with a Charlson Comorbidity Index > 2 than the patients who were included in the final analysis

	Lost to follow-up (*n* = 51)	*p*	Followed-up (*n* = 58)	*p*	Deceased (*n* = 71)
Age:	68.4 ± 16.4 (27–97) y	*0.800*	67.6 ± 16.2 (29–98) y	***< 0.001***	83.9 ± 11.3 (48–99) y
Sex	56.9% (29/51)	***0.021***	77.6% (45/58)	*0.358*	70.4% (50/71)
CCI > 2:	5.9% (3/51)	*0.544*	3.4% (2/58)	***< 0.001***	35.2% (25/71)
NOT	56.9% (29/51)	*0.370*	48.3% (28/58)	*0.085*	63.4% (45/71)
Follow-up:	10.2 ± 3.4 (6–17) y	*0.873*	10.3 ± 3.4 (5–17) y	*0.672*	10.5 ± 3.4 (5–16) y

The p values refer to the group comparison of the two adjacent groups on the right and left. Significant differences are expressed in bold numbers

Consequently, the final outcome analysis included 58 patients (45 females) with a mean age of 67.6 ± 16.2 years (range 29–98 years; males: 56.4 ± 14.7y, females: 70.9 ± 15.3y) at the time of injury. Of these, 30 patients underwent treatment with intramedullary nailing (IMN), while 28 patients were treated non-operatively (NOT). The mean follow-up duration for these 58 patients was 10.3 ± 3.4 years (range 5–17 years), and the follow-up period was longer for patients who received IMN (9.5 ± 2.8 years vs. 11.0 ± 3.7 years; *p* = .046). Patient age and follow-up were normally distributed in our population (*p* > .05). Patient characteristics did not significantly differ between the two treatment groups concerning age at time of injury (NOT: 64.8 ± 16.9 years; IMN: 70.2 ± 15.4years; *p* = .874), sex (NOT: 78.6% female; IMN: 76.7% female; *p* = .862), the proportion of serious comorbidities (CCI > 2; NOT: 1/28; IMN 1/30; *p* = .960), or additional injuries to the affected arm (NOT: 4/28 patients; IMN: 8/30 patients; *p* = .245). In both treatment groups, patients were most frequently treated for 3-part fractures including the greater tuberosity (Neer type IV). The proportion of Neer type V fractures was slightly higher in the IMN group, but the difference was not statistically significant (*p* > .05) (see Table 2).

**Table 2:** Distribution of fracture types in the treatments groups

NOT					IMN				
	Codman’s key fragments					Codman’s Key fragments			
	**2-part**	**3-part**	**4-part**	**Σ**		**2-part**	**3-part**	**4-part**	**Σ**
Neer III	6			6	**Neer III**	4			4
Neer IV		15	4	19	**Neer IV**		17		17
Neer V			3	3	**Neer V**			9	9
Σ	6	15	7	**28**	**Σ**	4	17	9	**30**

Notably no Neer type IV/2 and V/2 fractures were included in both groups due to their lacking indication for IMN

### Long term functional results of displaced PHF (Neer types III, IV, V)

**Fig. 3:**
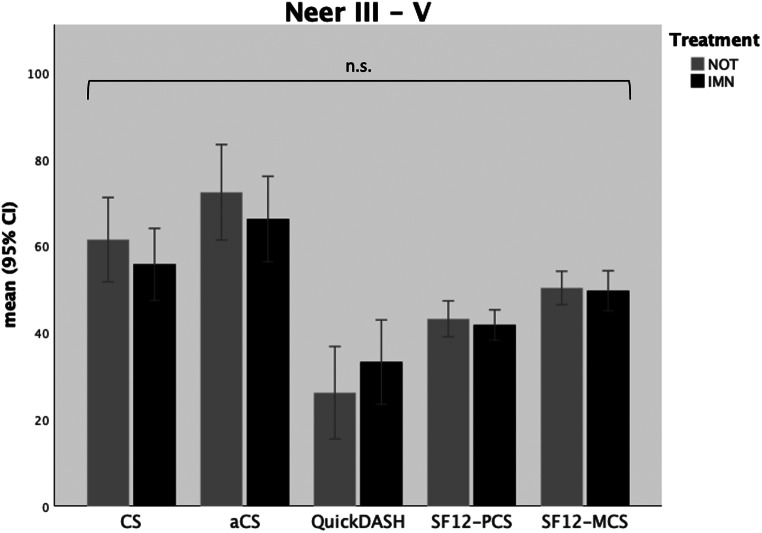
Functional outcome scores in overall population; IMN = intramedullary nailing; NOT = non-operative treatment; CS = Constant (Murley) Score; aCS = adapted Constant (Murley) Score; SF12-PCS = Short-Form 12 Physical Health Component Score; SF12-MCS = Short-Form 12 Mental Health Component Score

Analyzing the whole final study cohort, the reported functional outcomes, as indicated by the mean Constant score (CS) of 59.9 ± 22.9 points, remained compromised after the long-term period. However, the age- and sex-adapted Constant Score (aCS) demonstrated markedly improved overall functional outcomes, characterized as satisfactory (mean aCS 70.6 ± 26.0 points). There were no statistically significant differences in functional outcomes between the non-operative group and the intramedullary nailing group (see Fig. 3) when assessing both Constant scores (*p* = .350) and adapted Constant scores (*p* = .438) across all included patients. Disabilities of the affected arm in daily life, as measured by the QuickDASH score, persisted with an average score of 28.3 ± 25.1 points but did again not differ significantly between patients treated by IMN or NOT (*p* = .374). Similarly, quality of life in terms of both physical health (*p* = .594) and mental health (*p* = .820) did not significantly differ between the groups (see Table 3a).

**Table 3a:** Minimum 5-year postoperative outcome scores of patients treated either by non-operative therapy (NOT) or intramedullary nailing (IMN) did not show any statistically significant differences between the treatment groups

	Treatment		
	NOT	IMN	
	mean ± SD	mean ± SD	**p**
**CS**	62.8 ± 25.2	57.2 ± 20.6	*0.350*
**aCS**	73.6 ± 28.1	67.8 ± 24.0	*0.438*
**QuickDASH**	25.2 ± 26.9	31.1 ± 23.3	*0.374*
**SF-36-PCS**	43.2 ± 10.5	41.8 ± 8.7	*0.594*
**SF-36-MCS**	50.3 ± 9.7	49.7 ± 11.5	*0.820*

CS = Constant-Score; aCS = age- and sex-adapted Constant-Score; SF-36-PCS = Short Form 36, Physical Component Score; SF-36-MCS =  Short Form 36, Mental Component score

**Table 3b:** Minimum 5-year postoperative outcome scores of patients treated either by non-operative therapy (NOT) or intramedullary nailing (IMN) categorized by fracture types according to the neer classification system of proximal humerus fractures. There was no statistically significant difference between the treatment groups in any of the fracture types

	Treatment		
	NOT	IMN	
	mean ± SD	mean ± SD	**p**
**Neer type III**			
**CS**	72.8 ± 28.6	62.8 ± 16.5	*0.545*
**aCS**	84.8 ± 31.2	76.2 ± 20.9	*0.643*
**QuickDASH**	20.7 ± 26.2	22.2 ± 15.9	*0.925*
**SF-36-PCS**	47.2 ± 9.6	49.8 ± 6.4	*0.692*
**SF-36-MCS**	55.4 ± 7.1	59.7 ± 0.1	*0.338*
**Neer type IV**			
**CS**	61.0 ± 25.1	57.9 ± 21.2	*0.699*
**aCS**	71.3 ± 28.0	68.4 ± 25.2	*0.748*
**QuickDASH**	24.6 ± 27.2	30.9 ± 23.4	*0.467*
**SF-36-PCS**	41.5 ± 10.7	40.1 ± 8.4	*0.690*
**SF-36-MCS**	49.1 ± 10.7	47.0 ± 11.5	*0.583*
**Neer type V**			
**CS**	54.8 ± 21.3	53.3 ± 22.5	*0.919*
**aCS**	65.7 ± 25.1	62.9 ± 24.3	*0.870*
**QuickDASH**	38.0 ± 33.2	35.6 ± 26.5	*0.923*
**SF-36-PCS**	45.5 ± 11.7	42.1 ± 9.2	*0.632*
**SF-36-MCS**	47.5 ± 6.4	51.4 ± 12.0	*0.617*

CS = Constant-Score; aCS = age- and sex-adapted Constant-Score; SF-36-PCS = Short Form 36, Physical Component Score; SF-36-MCS = Short Form 36, Mental Component score

Irrespective of the chosen treatment modality, there was a trend towards poorer functional outcomes and increased limitations in daily activities with a higher number of Codman’s key fragments. In comparison to patients with 3- or 4-part fractures, those with only two key fragments (2-part fractures) exhibited significantly more favorable functional outcomes, as assessed by CS (2-part: 72.9 ± 21.8 points; > 2-part: 55.8 ± 21.9 points; *p* = .014), aCS (2-part: 85.0 ± 23.8 points; > 2 part: 66.0 ± 25.2 points; *p* = .015) and quality of life measured by SF-12-PCS (2-part: 48.5 ± 7.7 points; > 2-part: 40.8 ± 9.4 points; *p* = .012 and SF-12-MCS (2-part: 57.7 ± 5.5 points; > 2-part: 47.7 ± 10.6 points; *p* < .001). However, impairments in the activities of daily living, measured by the QuickDASH score, did not show a significant difference (2-part: 17.8 ± 19.8 points; > 2-part: 31.6 ± 25.8 points; *p* = .073).

### Subgroup analysis of fracture types

#### Neer type III fractures

**Fig. 4:**
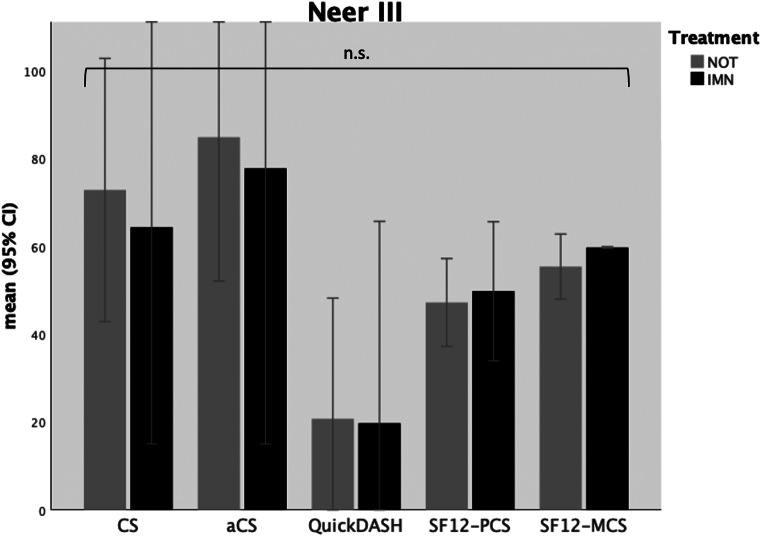
Functional outcome scores in patients with Neer type III fractures; IMN = intramedullary nailing; NOT = non-operative treatment; CS = Constant (Murley) Score; aCS = adapted Constant (Murley) Score; SF12-PCS = Short-Form 12 Physical Health Component Score; SF12-MCS = Short-Form 12 Mental Health Component Score

In 10 patients with Neer type III fractures (surgical neck fractures), both CS and aCS exhibited a tendency toward better function after non-operative treatment (NOT) compared to intramedullary nailing (IMN) but failed to reach statistical significance (CS: *p* = .545; aCS: *p* = .643, see Table 3b). The performance of daily activities without major impairments was observed in both treatment groups after Neer type III fractures (*p* = .925). Consequently, the quality of life was comparable in both groups (see Table 3b and Fig. 4). All surgically treated patients and 5 out of 6 patients in the NOT group achieved active abduction (aABD) and active forward flexion (aFF) greater than 90°.

#### Neer type IV fractures

**Fig. 5:**
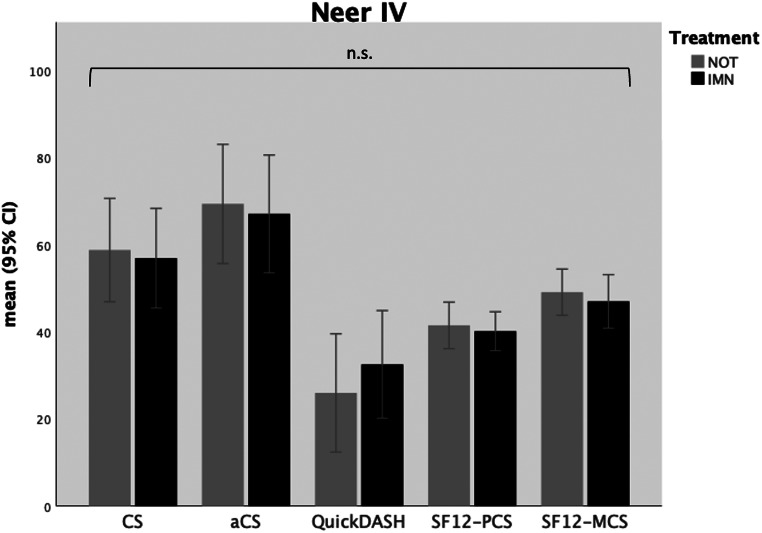
Functional outcome scores in patients with Neer type IV fractures; IMN = intramedullary nailing; NOT = non-operative treatment; CS = Constant (Murley) Score; aCS = adapted Constant (Murley) Score; SF12-PCS = Short-Form 12 Physical Health Component Score; SF12-MCS = Short-Form 12 Mental Health Component Score

In 36 Neer type IV fractures, both the CS (*p* = .699) and aCS (*p* = .748) did not exhibit significant differences between the treatment groups (see Table 3b). However, there was a statistically non-significant tendency towards fewer impairments in the QuickDASH questionnaire after non-operative treatment compared to treatment by nailing (*p* = .467). Furthermore, there were no significant differences in the SF-12-PCS (*p* = .690) and SF-12-MCS (*p* = .583) (see Table 3b and Fig. 5). Both aABD and aFF over 90° were achieved by 73.7% after NOT and 88.2% after IMN (*p* = .365).

#### Neer type V fractures

**Fig. 6:**
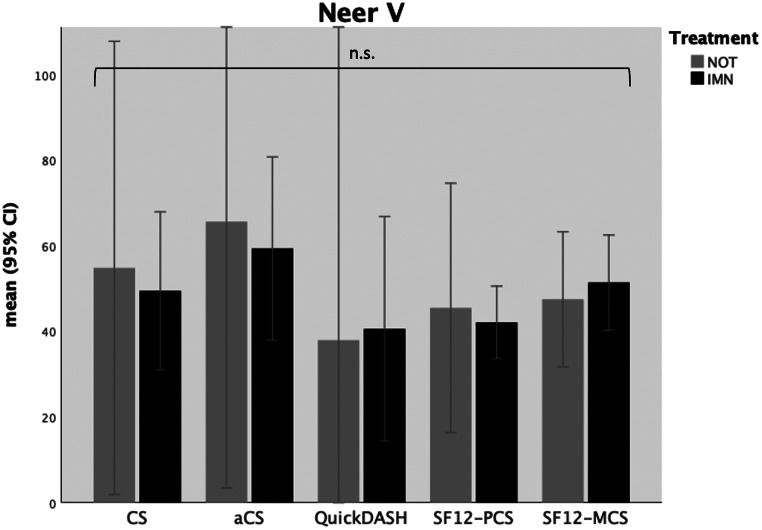
Functional outcome scores in patients with Neer type V fractures; IMN = intramedullary nailing; NOT = non-operative treatment; CS = Constant (Murley) Score; aCS = adapted Constant (Murley) Score; SF12-PCS = Short-Form 12 Physical Health Component Score; SF12-MCS = Short-Form 12 Mental Health Component Score

In 12 Neer type V fractures, there were no significant differences between both treatment groups regarding the outcome measurements by CS (*p* = .919), aCS (*p* = .870), SF-12-PCS (*p* = .632) and SF-12-MCS (*p* = .617). Furthermore, disabilities of the arm, shoulder and hand (QuickDASH) were also comparable between the treatment groups (*p* = .923) (see Table 3b and Fig. 6).

#### Complication rate and revision surgeries

In the cohort of 58 patients analyzed, revision surgery was required for 16 patients, all belonging to the IMN group, while an additional 3 patients encountered complications without necessitating surgical intervention (1 in the IMN group, 2 in the NOT group). Notably, none of the patients in the non-operative group underwent revision surgery or received total shoulder arthroplasty for posttraumatic osteoarthritis during the follow-up period. One patient in this group experienced restricted range of motion, managed nonoperatively through intensified physiotherapy.

In contrast, within the IMN group, 16 out of 30 patients underwent one or more additional surgeries after the primary IMN implantation. Among these, 6 patients underwent simple implant removal of the intramedullary nail due to restricted motion or soft tissue irritation. However, 10 out of 30 patients required further procedures to address more severe complications (see Table 4): 5 patients underwent surgical arthrolysis (2 open, 3 arthroscopic) to address postoperative shoulder stiffness, 2 patients required rotator cuff repair in conjunction with hardware removal due to anterosuperior rotator cuff tears at the nail entry site, 2 patients underwent nail removal and re-osteosynthesis using a proximal humeral locking-plate following secondary dislocation of fragments (1 patient) or refracture of the humeral head during implant removal (1 patient). Notably, only one patient was converted to reverse total shoulder arthroplasty due to the secondary dislocation of key fragments.

**Table 4:** Revision surgeries during follow-up period

non-operative treatment (*n* = 28)	intramedullary nailing (*n* = 30)	*p*
**without revision surgery (n = 28)**	•**without revision surgery (n = 14)**	< 0.001*
	•**removal of intramedullary nail (n = 6)**	
	•**removal of intramedullary nail ** **+ additional procedure (n = 10)**	
	– arthroscopic arthrolysis (*n* = 3)	
	– open arthrolysis (*n* = 2)	
	– rotator cuff repair (*n* = 2)	
	– re-osteosynthesis by LPF (*n* = 2)	
	– conversion to rTSA (*n* = 1)	

LPF = Locking Plate Fixation; rTSA = Reverse Total Shoulder Arthroplasty; Statistically significant differences in the distribution of patient characteristics are indicated by an asterisk (*)

The revision rate remained unaffected by the fracture classification according to Neer, with rates of 50.0% in Neer type III, 52.9% in Neer type IV, and 55.6% in Neer type V (*p* = .982).

## Discussion

This study sought to add to the existing literature through an observational investigation comparing the long-term outcomes of intramedullary nailing for proximal humeral fractures to non-operative treatment.

The key findings of this trial were:

- Comparing intramedullary nailing to non-operative treatment of Neer type III, IV and V proximal humerus fractures, there was no significant difference in long-term functional outcome and health related quality of life neither in the whole cohort nor in any subgroup.
- IMN seems to have a certain advantage in regaining active range of motion in the long term especially in fractures including the greater tuberosity (Neer type IV), but differences were not statistically significant.
- Revision rates were significantly elevated in the surgically treated patient group. One third of patients treated by IMN for PHFs underwent revision surgery that exceeded removal of the nail alone.

Our long-term study findings seamlessly integrate into the existing short- to mid-term literature [7, 13], suggesting comparable functional outcome of non-operative treatment to intramedullary nailing even in the long-term interval in a general population including all fracture types. The presented data, thus, highlight the value of non-operative treatment even in dislocated fractures.

In a comparable cohort study, Lange et al. [13] investigated the clinical and radiological outcomes of intramedullary nailing (IMN) and non-operative treatment (NOT) in 2-, 3-, and 4-part fractures with a minimum follow-up of 12 months. They observed an improved radiological outcome after IMN compared to NOT, although this improvement was not reflected in the functional results [13]. Similarly, in our long-term data, no significant benefits from operative treatment with IMN were found regarding functional outcomes measured by Constant Score, QuickDASH, and SF-12 after a mean follow-up of 10 years. Both treatment groups exhibited satisfactory outcome and function in daily activities. Though, a remaining limitation of active range of motion was observed in 26% of the NOT patients with fractures involving a displacement of the greater tuberosity (Neer Type IV) was observed. In contrast, the proportion of patients suffering from limited range of motion with less than 90° of active abduction was only half as high (12%) in the IMN group. This observation may be associated with a more stable retention of key fragments and a lower rate of secondary dislocation of the tuberosities in operatively treated patients, leading to lower rates of subacromial impingement and secondary rotator cuff lesions. This aligns with the observation of improved radiological results after operative treatment in the study by Lange et al. [13]. Emphasizing the importance of tuberosity position on long-term range of motion, Ge et al. observed improved external rotation at 6, 12, and 24 months after surgical treatment compared with NOT for fractures with displaced tuberosities [14]. In this trial, functional outcome scores for 2-part fractures did not show improvement with surgical treatment within the first two years, but, in 3-part fractures surgical treatment significantly enhanced functional scores at all follow-up visits [14]. Consistently, a meta-analysis by Sabharwal et al. [15] revealed that higher complexity fractures, including those with displaced tuberosities, such as 4-part fractures, derive the greatest benefit from surgical intervention.

On the contrary, the outcomes of several substantial randomized trials, such as the ProFHER trial [2], and recent meta-analyses [15, 16], have triggered a resurgence in the adoption of non-operative treatment. The influential ProFHER trial, published in 2015, compared surgical versus non-operative approaches in 250 displaced humerus fractures involving the surgical neck. The evaluation of functional outcomes revealed no significant differences at 6, 12, or 24 months [2] and even in a prolonged assessment spanning a total of 5 postoperative years [17]. Another randomized controlled trial by Launonen et al. [18] compared non-operative treatment with surgical treatment for 2-part fractures of the surgical or anatomical neck in patients aged 60 years or older. The authors found comparable functional results in both groups at 3 months, 6 months, 1 year, and two years, with minimal impairments in the activities of daily living (DASH 17.4 points in the non-operative group) [18]. Furthermore, a recent Cochrane Database Systematic Review encompassing 47 randomized and quasi-randomized trials by Handoll et al. [16] indicated no functional advantage of any surgical treatment over non-operative therapy for displaced humeral fractures in the initial two years following injury [16]. Limiting, most of the available studies comparing head preserving surgical therapy with non-operative treatment include locking plate fixation and not intramedullary nailing. Notably, only 4 of 109 patients (3.7%) in the surgical group received intramedullary nailing within the ProFHER trial, as the mode of surgical treatment was chosen by the treating surgeons and the majority was treated by locking plate fixation [2]. Nevertheless, most studies on this topic consistently report an elevated rate of complications and subsequent surgeries after surgical treatment of proximal humerus fractures, particularly after head-preserving surgery [2, 10, 13, 18]. These observations can be confirmed by our long-term study results.

Significantly, the surgical method compared to non-operative treatment in this study is not the preferred treatment option for most orthopedic surgeons treating patients in this age group (mean age over 65 years), as primary reverse arthroplasty is increasingly utilized [6] in the elderly population due to improved results when compared to delayed arthroplasty [19]. However, we selected intramedullary nailing as a minimally invasive, head-preserving surgical option for comparison with non-operative treatment. Our aim was to investigate whether the declining number of intramedullary nailing procedures can be supported by long-term results. It is important to emphasize that the study was not intended to discourage the use of intramedullary nailing. Instead, the focus was on highlighting the importance of determining the optimal treatment for each patient based on evidence-based treatment algorithms.

Considering all the factors discussed above, both intramedullary nailing and conservative treatment remain valuable strategies in the personalized management of proximal humerus fractures. Treatment decisions must be based on comprehensive evaluation of various fracture-related (such as the number of key fragments [20], varus/valgus dislocation [21], and bone quality [20, 22, 23]) and patient-related factors (including age [20, 24, 25], comorbidities [24], functional demands [20]). Treatment algorithms can guide these decisions. Spross et al. [20] introduced an algorithmic approach grounded in existing evidence. According to their algorithm, over 80% of the patients in the study could be effectively treated non-operatively [20], and algorithm-guided treatment demonstrated significantly superior outcomes in terms of function, complication rates, and revision rates compared to approaches not aligned with the algorithm [26]. Interestingly, possibly due to limited long-term evidence, intramedullary nailing was not included as a treatment option in the algorithm.

Our current investigation is subject to several limitations, primarily associated with the retrospective nature of the analysis, such as selection bias. Although the patients in our cohorts did not differ significantly in terms of the proportion of patients with severe comorbidities, there could be a potential selection bias in favor of sicker patients receiving non-surgical treatment. Overall, indications for surgical or non-operative treatment of the study patients are not standardized in this study given its retrospective nature. Although surgeons’ preferences are known to highly impact treatment choice in clinical routine [27, 28], treatment decisions in our trauma center were primarily influenced by evidence-based factors such as age, comorbidities, and the specific fracture characteristics such as translation type displacement and comminution of the medial hinge. Nevertheless, other factors like associated orthopaedic injuries requiring surgery, and patients’ preferences and functional demands likely also influenced treatment choices.

Given that proximal humerus fractures are common in the elderly population, mid- to long-term mortality rates are known to be high following these injuries [29]. As anticipated, our analysis revealed a substantial number of deceased patients over the 10-year follow-up period, significantly impacting the remaining cohort sizes available for functional evaluation. Nevertheless, including deceased patients, the overall high rate of available follow-up data (129/180 patients, 72%) marks a highlight of the trial. As the cohort of deceased patients was significantly older (mean 83.9 years) and had a higher proportion of seriously comorbid patients, the applicability of the study results to this vulnerable patient group is limited.

Moreover, assessment of the functional outcome and range of motion was not performed in person but via telephone interviews, which entails the risk of a less accurate assessment but increases follow-up rate. In order to improve validity of the self-evaluated outcome parameters, telephone interviews were conducted by orthopedic professionals and an adapted self-assessment version of the Constant Score [12] was used.

Lastly, since the follow-up surveys were largely collected via telephone interviews no radiographic data was included. However, the aim of the present study was rather to present the long-term outcome after intramedullary nailing than investigating the radiologic outcome or the influence of the quality of surgical care in shortly postoperative radiographs. Although reduction of the humeral head, medial hinge, and tuberosities is believed to directly impact functional and patient-reported subjective outcomes in some studies [30], Lange et al. [13] described that healing with or without displacement after intramedullary nailing of PHF had no influence on the average side-adapted Constant score in their study. Additionally, since patients were followed up after a mean period of 10 years in our trial, we assume that poor reduction or the occurrence of AVN is displayed as limited range of motion, limited function, or the need for secondary revision surgery.

## Conclusion

In our investigation of long-term outcomes, patient-reported outcome measures revealed no significant differences between intramedullary nailing and non-operative treatment for Neer type III–V proximal humerus fractures while, at the same time, operative treatment led to an increased rate of secondary surgery. Consequently, this study underlines that intramedullary nailing does not provide a clinically significant benefit in patients where open reduction and internal fixation or arthroplasty are deemed less favorable due to increased invasiveness and demanding aftercare. In this group of patients, non-operative treatment should always be considered in order to avoid the need for secondary surgery. On the other hand, cases for intramedullary nailing must be carefully selected.

The expanding literature underscores the critical role of customizing treatment decisions based on patient and injury factors for each patient.

### Declaration of generative AI and AI-assisted technologies in the writing process

During the preparation of this work the author(s) used ChatGPT in order to improve language and readability. After using this tool/service, the author(s) reviewed and edited the content as needed and take(s) full responsibility for the content of the publication.
